# GTMALoc: prediction of miRNA subcellular localization based on graph transformer and multi-head attention mechanism

**DOI:** 10.3389/fgene.2025.1623008

**Published:** 2025-06-19

**Authors:** Xindi Huang, Jipu Jiang, Lifen Shi, Cheng Yan

**Affiliations:** School of Informatics, Hunan University of Chinese Medicine, Changsha, China

**Keywords:** miRNA, subcellular localization, graph transformer, multi-head attention mechanism, multi-source features

## Abstract

MicroRNAs (miRNAs) play a crucial role in regulating gene expression, and their subcellular localization is essential for understanding their biological functions. However, accurately predicting miRNA subcellular localization remains a challenging task due to their short sequences, complex structures, and diverse functions. To improve prediction accuracy, this study proposes a novel model based on a graph transformer and a multi-head attention mechanism. The model integrates multi-source features which include the miRNA sequence similarity network, miRNA functional similarity network, miRNA–mRNA association network, miRNA–drug association network, and miRNA–disease association network. Specifically, we first apply the node2vec algorithm to extract features from these biological networks. Then, we use a graph transformer to capture relationships between nodes within the networks, enabling a better understanding of miRNA functions across different biological contexts. Next, a multi-head attention mechanism is implemented to combine miRNA features from multiple networks, allowing the model to capture deeper feature relationships and enhance prediction performance. Performance evaluation shows that the proposed method achieves significant improvements over current approaches on open-access datasets, achieving high performance with an AUC (area of receiver operating characteristic curve) of 0.9108 and AUPR(area of precision-recall curve) of 0.8102. It not only significantly improves prediction accuracy but also exhibits strong generalization and stability.

## 1 Introduction

MicroRNAs (miRNAs) are a class of small non-coding RNAs widely distributed in eukaryotic cells, typically around 22 nucleotides in length. They mainly regulate gene expression through the post-transcriptional processes Hombach and Kretz (2016); Holley and Topkara (2011). In organisms, miRNAs bind to specific target sites on mRNAs, causing their subsequent degradation or translational inhibition, thereby modulating key fundamental physiological processes like cell proliferation, differentiation, apoptosis, and immune system activation Bartel (2009). Recent studies have shown that miRNAs have indispensable functions in a variety of human diseases, including cancer, neurodegenerative disorders, and cardiovascular diseases Li et al. (2023); Dugger and Dickson (2017). They also show great potential in drug response prediction, resistance mechanisms, and therapeutic target discovery Miska (2007); Small and Olson (2011). In this context, studying the subcellular localization of miRNAs is of great significance for understanding their regulatory networks and functional mechanisms Kabekkodu et al. (2018); Catalanotto et al. (2016); Gurtan and Sharp (2013). Different subcellular localizations often suggest that miRNAs are involved in distinct biological processes. Accurate localization prediction not only facilitates the understanding of functional diversification of miRNAs but also provides theoretical support for early disease diagnosis and targeted therapy Jie et al. (2021). Although conventional experimental methods, such as fluorescence *in situ* hybridization and subcellular fractionation combined with high-throughput sequencing, can directly determine miRNA distributions, these techniques are often complex, expensive, and lack scalability for large-scale samples Thomson and Dinger (2016). Therefore, developing computational methods to efficiently predict miRNA subcellular localization has become a focal point in bioinformatics research. Currently, researchers have developed various machine learning models based on sequence information to explore potential miRNA localization patterns. For example, Huang et al. (2007) proposed a prediction framework combining k-mer frequency patterns with a Support Vector Machine (SVM) classifier, showing the feasibility of using sequence information for localization recognition. However, due to the short length and complex structure of miRNAs, as well as their heterogeneity across different tissues or disease states, models relying solely on sequence-level features often fail to capture the complete biological semantics, resulting in limited accuracy and generalization Li et al. (2014). To address this, some studies have incorporated biological network information, such as the miRNA-mRNA interaction network Hsu et al. (2011), the miRNA-disease association network Jiang et al. (2010), and the miRNA-drug association network Chen H. et al. (2019), to improve prediction accuracy. For instance, Xie et al. constructed a miRNA-target gene interaction network using Graph Convolutional Networks (GCNs) and applied deep learning to predict miRNA functions within cells Guan et al. (2022). Li et al. integrated miRNA, disease, and drug information through a heterogeneous network and used graph embedding techniques for feature learning Sun et al. (2020). Over the past few years, deep learning innovations have significantly advanced bioinformatics research. Convolutional Neural Networks (CNNs) have been used to retrieve sequence features of miRNAs—such as in the DeepMirTar model, which utilized CNNs to improve target gene prediction accuracy Wen et al. (2018). Recurrent Neural Networks (RNNs) have also been employed to capture sequential dependencies, as seen in MirLocNet, which uses Long Short-Term Memory (LSTM) networks to computationally infer miRNA subcellular localization Chen Q. et al. (2019). Graph Neural Networks (GNNs) are widely applied in modeling biological networks. As proposed by Gao et al. (2022), a novel Graph Attention Networks (GATs) combined with biological network information to improve miRNA function prediction Zhao et al. (2022).

In the field of miRNA subcellular localization, researchers have developed various models to enhance prediction accuracy and biological interpretability. MiRLoc Xu et al. (2022), for instance, inferred miRNA spatial distribution by leveraging known mRNA localization and their interaction with miRNAs, reflecting their role in post-transcriptional regulation. MirLocPredictor Asim et al. (2020) incorporated CNNs and positional encoding of k-mers to enhance sequence representation for multi-label localization tasks. DAmirLocGNet Bai et al. (2023a) integrated Graph Convolutional Networks and autoencoders to jointly model miRNA sequence features, disease associations, and disease semantic networks, learning high-level representations from complex graph structures. Some existing excellent models provide us with references. For example, Wang X.-F. et al. (2024) proposed a multi-channel graph neural network framework that integrates multimodal similarity information with hypergraph contrastive learning, effectively identifying novel cancer biomarkers. Wang X.- et al. (2024) designed a directed graph neural network-based multi-view learning model capable of systematically extracting regulatory feature signals from multiple biological layers, enhancing the model’s representational power. Additionally, Wang et al. (2022) developed KGDCMI, a method that integrates multi-source biological information with deep learning techniques to accurately predict interactions between circRNA and miRNA. Comparatively, PMiSLocMF Chen et al. (2024) fused heterogeneous data such as miRNA-mRNA, miRNA-drug, and miRNA-disease networks using a graph attention mechanism, achieving robust performance even in scenarios with sparse data or incomplete labels. Despite improvements, present architectures still face challenges such as inadequate information integration and underutilization of multi-head feature relationships. Effectively integrating multi-source information and building more expressive feature representations to improve miRNA subcellular localization prediction remains an urgent and critical problem.

To overcome these limitations, this study proposes a novel miRNA subcellular localization prediction model named GTMALoc, based on graph transformer and multi-head attention mechanisms. This approach effectively incorporates miRNA sequence information and their roles across different biological networks to improve prediction performance. Specifically, we first extract miRNA features from multiple biological networks–including miRNA sequence similarity, miRNA-mRNA associations, miRNA-disease associations, and miRNA-drug associations—using node2vec. Then, a graph transformer framework is applied to infer latent node correlations, offering better insight into miRNA functionality in different contexts. A multi-head attention mechanism is subsequently employed to integrate miRNA features across networks, capturing deeper, multi-head relational patterns and enhancing predictive performance. The evaluations show that our model outperforms mainstream methods in terms of accuracy, generalization, and stability on public datasets, demonstrating its effectiveness and feasibility in the miRNA subcellular localization task. Key improvements over existing methods provided by this study are:

(1) We propose a new miRNA subcellular localization prediction model that leverages graph transformer and multi-head attention mechanisms to integrate multi-source biological network information.
(2) Complex relationships within biological networks are modeled using node2vec and graph transformer to improve high-dimensional representations of miRNA features.
(3) A multi-head attention mechanism is employed to fuse heterogeneous network information, thereby strengthening inter-feature relationships and improving the prediction accuracy and generalization ability of model.

## 2 Materials and methods

### 2.1 Datasets

The dataset used in this study is sourced from version 2.0 of the RNALocate database Cui et al. (2022), which systematically compiles a large number of experimentally support RNA subcellular localization records. From this database, we select a subset containing 1,041 miRNAs to construct and evaluate our model. To ensure biological consistency, all select miRNAs are included in the miRNA functional similarity network established in the MiRLoc Xu et al. (2022) study, facilitating the exploration of potential functional associations. In terms of localization annotation, these miRNAs are assigned to seven subcellular compartments: cytoplasm, nucleus, nucleolus, mitochondrion, exosome, microvesicle, and extracellular vesicle. The specific numbers are as follows: 870 exosomes, 825 microvesicle, 499 nucleus, 308 cytoplasms, 259 mitochondrion, 102 extracellular vesicle, and 67 nucleolus. This categorization not only covers the major cellular structures where miRNAs may reside but also reflects their diverse roles in intracellular and intercellular communication, providing a rich and challenging dataset for multi-label classification tasks.

### 2.2 Methods

**FIGURE 1:**
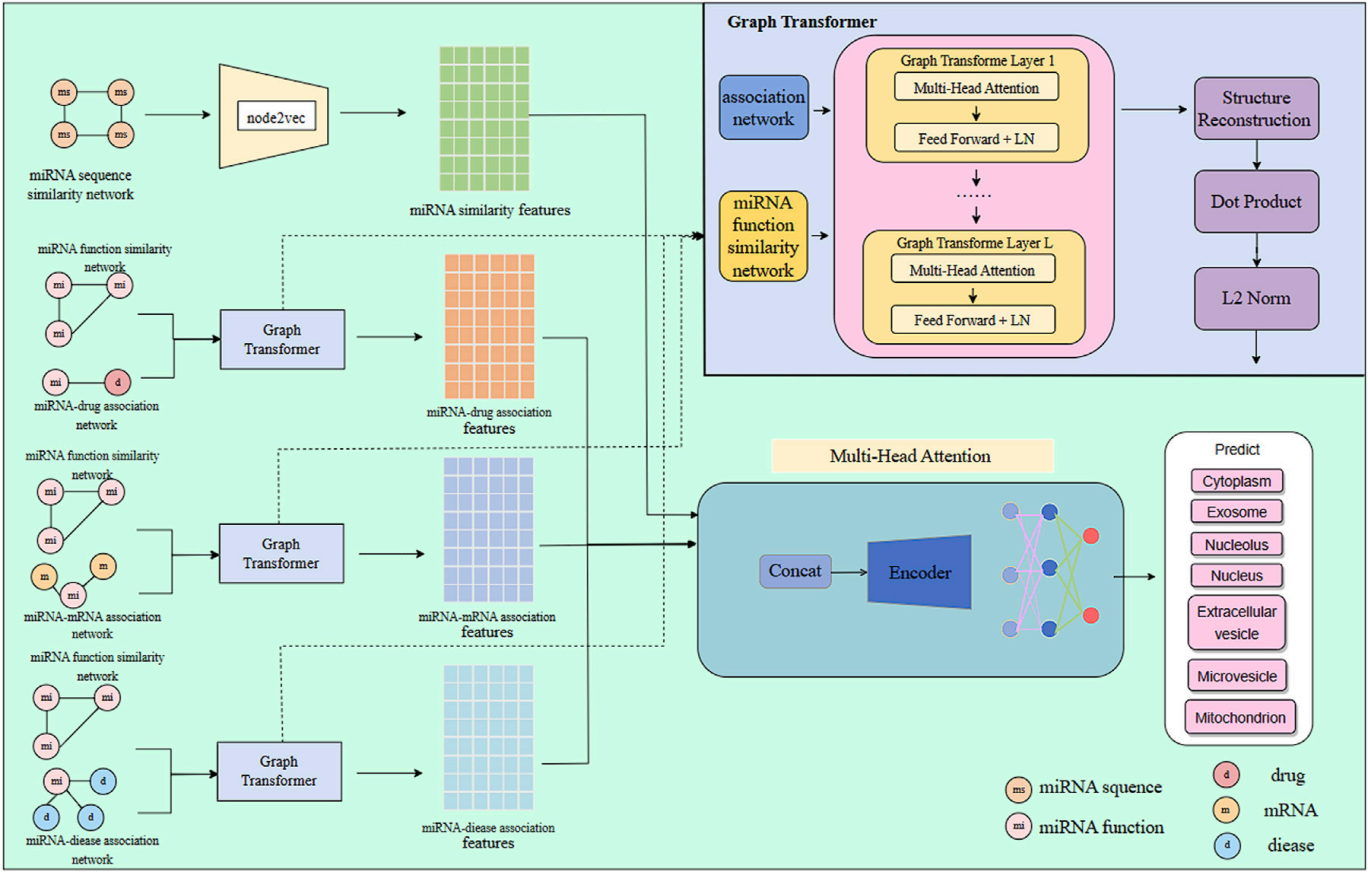
The architecture of the GTMALoc model.

In this study, we develop a multi-source feature fusion model, GTMALoc, for miRNA subcellular localization prediction, aiming to comprehensively capture miRNA characteristics in various biological contexts. This process is illustrated in Figure 1. First, we extract structural features from several biological networks, including the miRNA sequence similarity network, miRNA–mRNA regulatory network, miRNA–disease association network, and miRNA–drug interaction network. To preserve both local and global structural information within each network, we apply the node2vec algorithm to perform embedding learning on these heterogeneous graphs, thereby obtaining a representation vector for each miRNA under different semantic relations—reflecting its functional characteristics in diverse biological environments. Next, we utilize the graph transformer model to process the graph embedding features. Leveraging its built-in structural awareness and self-attention mechanism, the model captures complex and variable dependencies among nodes, enabling a deeper understanding of miRNA behavior and influence across different networks. To achieve effective multi-source information fusion, we further introduce a multi-head attention mechanism to align and integrate miRNA representations from various networks. This allows the model to automatically uncover important cross-network interactions and latent high-level semantic relationships. The fusion strategy not only enhances the model’s sensitivity to critical features but also significantly improves overall prediction accuracy and generalization performance.

## 3 miRNA networks

### 3.1 miRNA sequence similarity network and miRNA functional similarity network

All miRNA sequence data are obtained from the authoritative database miRBase (version 22) Kozomara et al. (2019), which provides experimentally validated miRNA sequences from humans and other species, and is widely used in miRNA research. To construct the miRNA sequence similarity network, we employ the Smith–Waterman algorithm Smith and Waterman, (1981), a classical local sequence alignment technique that precisely evaluates the similarity between two miRNA sequences in terms of base composition and order. Specifically, the algorithm uses dynamic programming to find optimal local alignments based on base matches, mismatches, and gap penalties, thereby computing a similarity score for each miRNA pair (Equation 1).

$$SW\left(m_{i},m_{j}\right)=\frac{sp\left(m_{i},m_{j}\right)}{\sqrt{sp\left(m_{i},m_{i}\right)\cdot sp\left(m_{j},m_{j}\right)}},$$ (1)

$sp\left(m_{i},m_{j}\right)$ represents the local alignment fraction of the two sequences, and a symmetrical similarity matrix can be obtained by performing the above alignment process between all miRNAs. The miRNA sequence similarity network was created according to the similarity matrix by assigning the miRNA as the node and the similarity score assigned to the corresponding edge as its weight. In order to develop a functional similarity network of miRNAs, we initially used the association data between miRNAs and diseases to construct a disease hierarchy with the help of medical subject headings (MeSH). Specifically, each disease $d_{i}$ is represented in an acyclic diagram (DAG) by a subgraph that includes the $d_{i}$ , and all of its higher-level diseases. For each disease $d_{t}$ in the subgraph, its contribution to $d_{i}$ can be expressed as (Equation 2):

$$C\left(d_{t},d_{i}\right)=a\times \frac{1}{\text{Depth}\left(d_{t},d_{i}\right)},$$ (2)

where  a  is the adjustment parameter, which $Depth\left(d_{t},d_{i}\right)$ represents the hierarchical distance between $d_{t}$ and $d_{i}$ . Next, the semantic value of disease $d_{i}$ is established by aggregating all node contributions within its subgraph (Equation 3):

$$SV\left(d_{i}\right)=\underset{d_{t}\in S\left(d_{i}\right)}{\sum }C\left(d_{t},d_{i}\right),$$ (3)

let $S\left(d_{i}\right)$ denote the set containing disease $d_{i}$ and all of its ancestor diseases. The semantic similarity between two diseases, $d_{i}$ and $d_{j}$ , denoted as $Sim\left(d_{i},d_{j}\right)$ , can be calculated based on the overlap of their semantic values, which reflects their semantic similarity. For two miRNAs, $m_{1}$ and $m_{2}$ , let their associated disease sets be $D_{1}$ and $D_{2}$ , respectively. Their initial functional similarity can then be defined as the average semantic similarity between diseases in $m_{1}$ and those in $m_{2}$ (Equation 4):

$$FS\left(m_{1},m_{2}\right)=\frac{1}{\left|D_{1}\cap D_{2}\right|}\underset{d\in D_{1}\cap D_{2}}{\sum }\text{Sim}\left(d\right).$$ (4)

The challenge of similarity underestimation arising from disease set sparsity is resolved through linear combination with miRNA GIP kernel similarity, generating robust functional similarity estimates (Equation 5).

$$FS^{*}\left(m_{1},m_{2}\right)=\lambda \;FS\left(m_{1},m_{2}\right)+\left(1-\lambda \right)\;GIP\left(m_{1},m_{2}\right),$$ (5)

where λ is a fusion parameter, and $GIP\left(m_{1},m_{2}\right)$ represents the similarity between the two miRNAs under the GIP kernel. Specifically, λ is used to balance the contributions of functional similarity and GIP kernel similarity. We perform a grid search over the values [0.1, 0.3, 0.5, 0.7, 0.9], using multi-label classification performance as the evaluation criteria. The optimal value is found to be λ = 0.5, which offered a good trade-off between generalization and robustness. For the threshold T used to binarize the similarity matrix, we adopt an empirical approach Wang et al. (2010), adjusting T to control the sparsity of the resulting adjacency matrix. We ultimately set T to 0.6 to ensure a reasonable balance between sparsity and connectivity in the resulting graph. After calculating the similarity between all miRNA pairs, a similarity matrix is constructed. This matrix is then binarized using a predefined threshold T (Equation 6):

$$A_{\text{ij}}=\left\{\begin{array}{cc} 1,\; & if\;FS^{*}\left(m_{i},m_{j}\right)>T\\ 0,\; & otherwise \end{array}\right..$$ (6)

The resulting adjacency matrix A defines the miRNA functional similarity network, where nodes represent miRNAs and edges reflect their functional similarity.

### 3.2 miRNA-mRNA association network

The miRNA–mRNA regulatory network in this study is primarily based on data from the authoritative miRTarBase (2020 version) Huang et al. (2020), supplemented by a curated dataset of validated interactions compiled by Xu et al. (2022). miRTarBase is known for its high-quality data, integrating miRNA–target gene interactions supported by both low- and high-throughput experimental evidence, such as reporter gene assays, qRT-PCR, and Western blot. The constructed network contains 8,254 high-confidence regulatory relationships between 1,041 non-coding miRNAs and 2,836 protein-coding genes.

### 3.3 miRNA-drug association network

The miRNA–drug association network is based on data from ncDR, a drug resistance research database Dai et al. (2017), which collects experimentally verified and predicted interactions between non-coding RNAs and drugs. The data are standardized as follows: First, the 1,041 miRNAs involved in previous studies are matched based on miRBase nomenclature. Second, only interactions with clearly annotated drug resistance evidence (including preclinical or cell line experiments) are retained. This results in 3,305 high-confidence miRNA–drug interactions involving 130 commonly used clinical drugs, such as cisplatin and gefitinib.

### 3.4 miRNA-diease association network

To construct the required network, this study references the dataset from HMDD v3.2 Bai et al. (2023b), a widely-used human microRNA disease database. After curation and filtering, 15,547 miRNA–disease association pairs are obtained, covering 1,041 miRNAs and 640 human diseases.

## 4 Node2vec algorithm

Network modeling has emerged as a pivotal paradigm in biomedical research due to its intuitive representation of complex relationships, particularly in systematic miRNA analysis involving multimodal correlations. This study integrates four critical biological networks: the miRNA sequence similarity network (quantifying functional conservation), the miRNA–disease association network (revealing pathological regulation), the miRNA–drug interaction network (reflecting therapeutic targeting), and the miRNA–mRNA regulatory network (decoding genetic circuitry). To effectively capture topological features from these non-Euclidean spatial data, we employ the node2vec algorithm Grover and Leskovec (2016), a graph embedding approach based on adaptive random walk strategies. By tuning search parameters—the return parameter p controlling local neighborhood sampling and the in-out parameter q governing global structural exploration—this approach generates semantically preserved node sequences, subsequently vectorized through Skip-Gram modeling. Notably, we implement dimension-specific embedding strategies tailored to distinct network characteristics: 64-dimensional representations in sequence similarity networks to resolve fine-grained patterns of conserved functional motifs, *versus* 128-dimensional high-capacity embeddings in the three heterogeneous association networks to capture complex multi-hop interactions. This hierarchical embedding mechanism simultaneously reduces feature redundancy while preserving network-specific information, establishing an interpretable mathematical foundation for subsequent multi-view feature fusion.

## 5 Graph transformer

This study proposes a structure-aware graph neural network, the graph transformer, to learn high-quality node embeddings from graph structures. Unlike traditional GNNs, which struggle with sparse or heterogeneous structures, the graph transformer incorporates multi-head attention and a structure reconstruction loss, enabling better modeling of local and global graph information. First, the input miRNA functional similarity matrix and association matrix are feature fused. After generating the node feature matrix $h_{k}$ at layer k , it dynamically rebuilds the attention weights matrix $a_{k+1}$ through dot products of $h_{k}$ and $h_{k}^{T}$ , enabling the joint evolution of topology and features. This gradient-preserving update critically suppresses false-positive edges caused by experimental noise. The model adopts Pre-Layer Normalization (Pre-LN), normalizing features before multi-head attention and feed-forward operations rather than after, which better accommodates high-dimensional biological feature propagation and curbs gradient vanishing in deep training. For extremely sparse data like miRNA-drug networks, binary attention masking embedded in multi-head layers automatically blocks unobserved associations (e.g., unknown miRNA-drug interactions), reducing computational complexity from $O\left(N^{2}\right)$ to O(E) (where E denotes valid edges) with less GPU memory consumption, while preventing noise from distorting attention weights. These improvements jointly optimize the model, and the output layer further sharpens the precision-recall curve through dot product similarity and L2 normalized feature constraints in the range of [0,1]. During embedding learning, the model stacks L layers of graph attention modules, where each node’s representation is updated by aggregating the representations of its neighbors (Equation 7):

$$h_{i}^{\left(l\right)}=\;\sigma \;\left(\underset{j\in N\left(i\right)}{\sum }A_{ij}^{\left(l\right)}W^{\left(l\right)}h_{j}^{\left(l-1\right)}\right).$$ (7)

Here, σ is an activation function, and $W^{\left(l\right)}$ is a learnable weight matrix. The attention weights $a_{ij}^{\left(l\right)}$ are calculated as (Equation 8):

$$a_{ij}^{\left(l\right)}=\frac{\exp\left(LeakyReLU\left(a^{⊤}\left[W^{\left(l\right)}h_{i}^{\left(l-1\right)}\|W^{\left(l\right)}h_{j}^{\left(l-1\right)}\right]\right)\right)}{\sum_{k\in N\left(i\right)}\exp\left(LeakyReLU\left(a^{⊤}\left[W^{\left(l\right)}h_{i}^{\left(l-1\right)}\|W^{\left(l\right)}h_{k}^{\left(l-1\right)}\right]\right)\right)},$$ (8)

where a is the attention weight vector, which ‖ represents the vector splicing operation. Graph transformer further introduces a multi-head attention mechanism, using K attention heads in parallel in each layer, and finally integrating their output splicing or averaging into the input of the next layer (Equation 9):

$$h_{i}^{\left(l\right)}=\frac{1}{K}\overset{K}{\underset{k=1}{\sum }}\sigma \left(\underset{j\in N\left(i\right)}{\sum }a_{ij}^{\left(l,k\right)}W^{\left(l,k\right)}h_{j}^{\left(l-1\right)}\right).$$ (9)

In order to enhance the model’s ability to express structural information, graph transformer also introduces structural reconstruction loss as the training goal. In the unsupervised setting, the model scores the node pairs of the real edges in the input graph, and defines the structural reconstruction similarity as (Equation 10):

$$s_{ij}=\sigma \left(h_{i}^{⊤}h_{j}\right),$$ (10)

where $h_{i},h_{j}$ are the final embeddings of nodes i and j, and  σ denotes the sigmoid function. The structural reconstruction loss is formulated as (Equation 11):

$$L_{\text{reconstruct}}=-\underset{\left(i,j\right)\in E^{+}}{\sum }\log\left(\sigma \left(h_{i}^{⊤}h_{j}\right)\right)-\underset{\left(i,j\right)\in E^{-}}{\sum }\log\left(1-\sigma \left(h_{i}^{⊤}h_{j}\right)\right),$$ (11)

where $E^{+}$ represents the set of true edges, and $E^{-}$ denotes negative-sampled non-edges to prevent degenerate solutions where all nodes become indistinguishable. This objective effectively preserves high separability of node connectivity patterns in the embedding space. The final node embeddings $H=\left[h_{1},h_{2},...,h_{N}\right]$ undergo L2-normalization for downstream tasks.

## 6 Multi-head attention mechanism

To capture cross-modal dependencies and interactions among various biological features, a Multi-Head Attention (MHA) module is introduced as the core feature interaction component in the fusion model. Based on the transformer encoder, MHA computes attention across multiple subspaces in parallel to enhance local and global correlation modeling. Four input feature types—miRNA sequence, drug features, mRNA features, and disease features are first projected to a common 128-dimensional space using fully connected layers with L2 regularization. These are concatenated and reshaped into a 2D sequence format before being passed into the MHA module. The Multi-Head Attention Mechanism is calculated as follows (Equations 12, 13):

$$Q=HW^{Q},\;K=HW^{K},\;V=HW^{V},$$ (12)

$$\text{Attention}\left(Q,K,V\right)=\text{softmax}\left(\frac{QK^{⊤}}{\sqrt{d_{k}}}\right)V.$$ (13)

The outputs of all heads are concatenated and transformed, the formula is as follows (Equations 14, 15):

$$MHA\left(H\right)=Concat\left({head}_{1},\ldots ,{head}_{h}\right)W^{0},$$ (14)

$$H^{\left(1\right)}=LayerNorm\left(H+Dropout\left(MHA\left(H\right)\right)\right).$$ (15)

To further improve the representation capability, the multi-head attention output will be delivered through two layers of Feed-Forward Network (FFN), the formula is as follows (Equations 16, 17):

$$FFN\left(H\right)=ReLU\left(HW_{1}+b_{1}\right)W_{2}+b_{2},$$ (16)

$$H_{\text{fusion}}=LayerNorm\left(H^{\left(1\right)}+Dropout\left(FFN\left(H^{\left(1\right)}\right)\right)\right).$$ (17)

The final output represents the fused multimodal semantic embedding features, which are used as the input of the subsequent self-supervised learning projection head and the multi-label classification header, which not only retains the information of the original modal features, but also integrates the high-order correlation between them.

## 7 Prediction of miRNA subcellular localization

During forward propagation, the fused high-dimensional features are processed through the MHA and FFN modules. Finally, the classification head maps the features to predicted subcellular localization probabilities (Equation 18):

$$\hat{y}=o\left(f_{\mathrm{cls}}\left(x\right)\right),$$ (18)

where $f_{\mathrm{cls}}$ is a linear mapping and  o  is the Sigmoid activation function that converts the output into a probability vector over [0, 1]. For each class, if the predicted probability exceeds the threshold (0.5), the class is labeled as positive; otherwise, negative–resulting in the final binary classification output.

## 8 Results

### 8.1 10-Fold cross-validation

**FIGURE 2:**
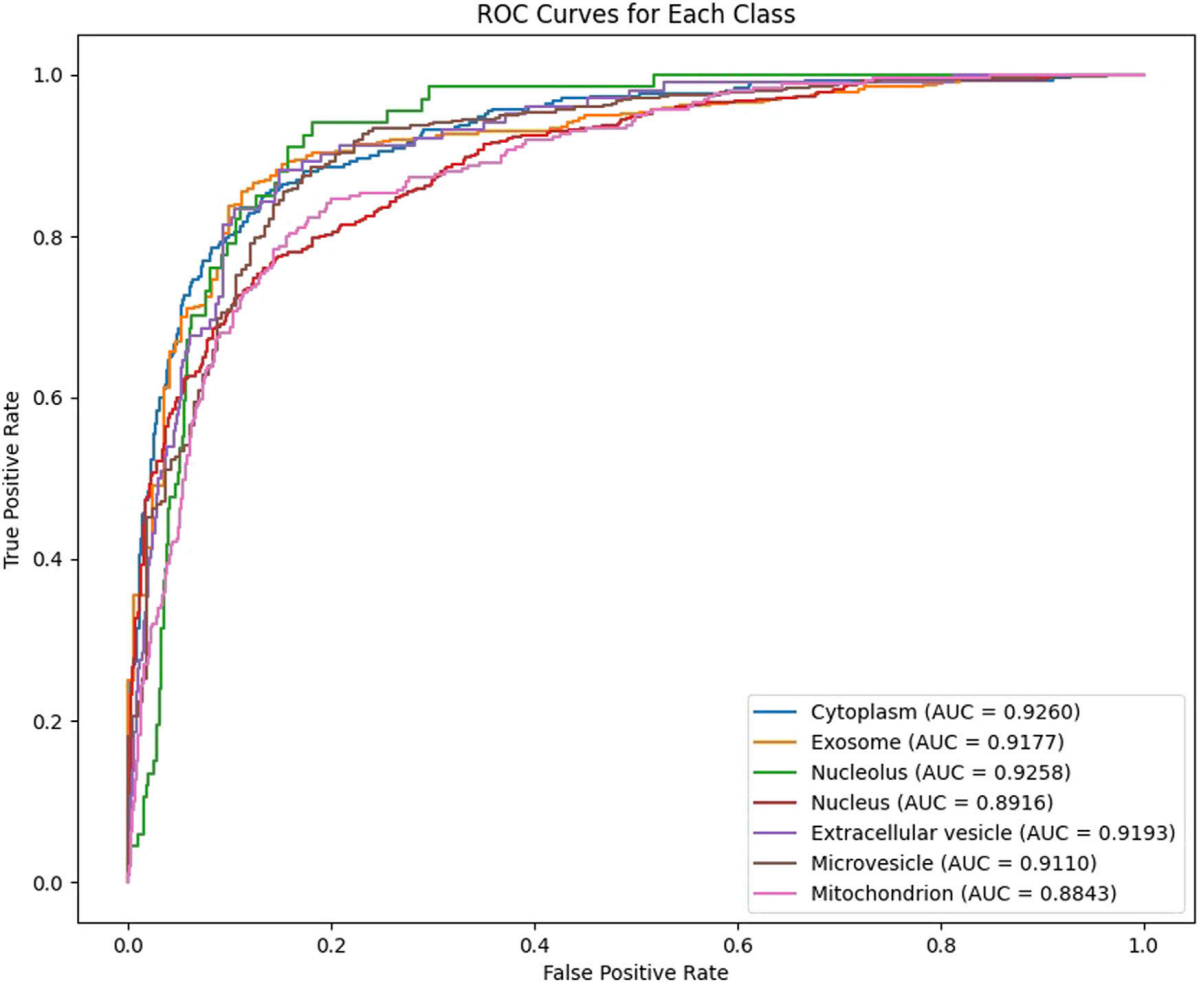
The Model GTMALoc 10-fold cross-validation AUC value.

**FIGURE 3:**
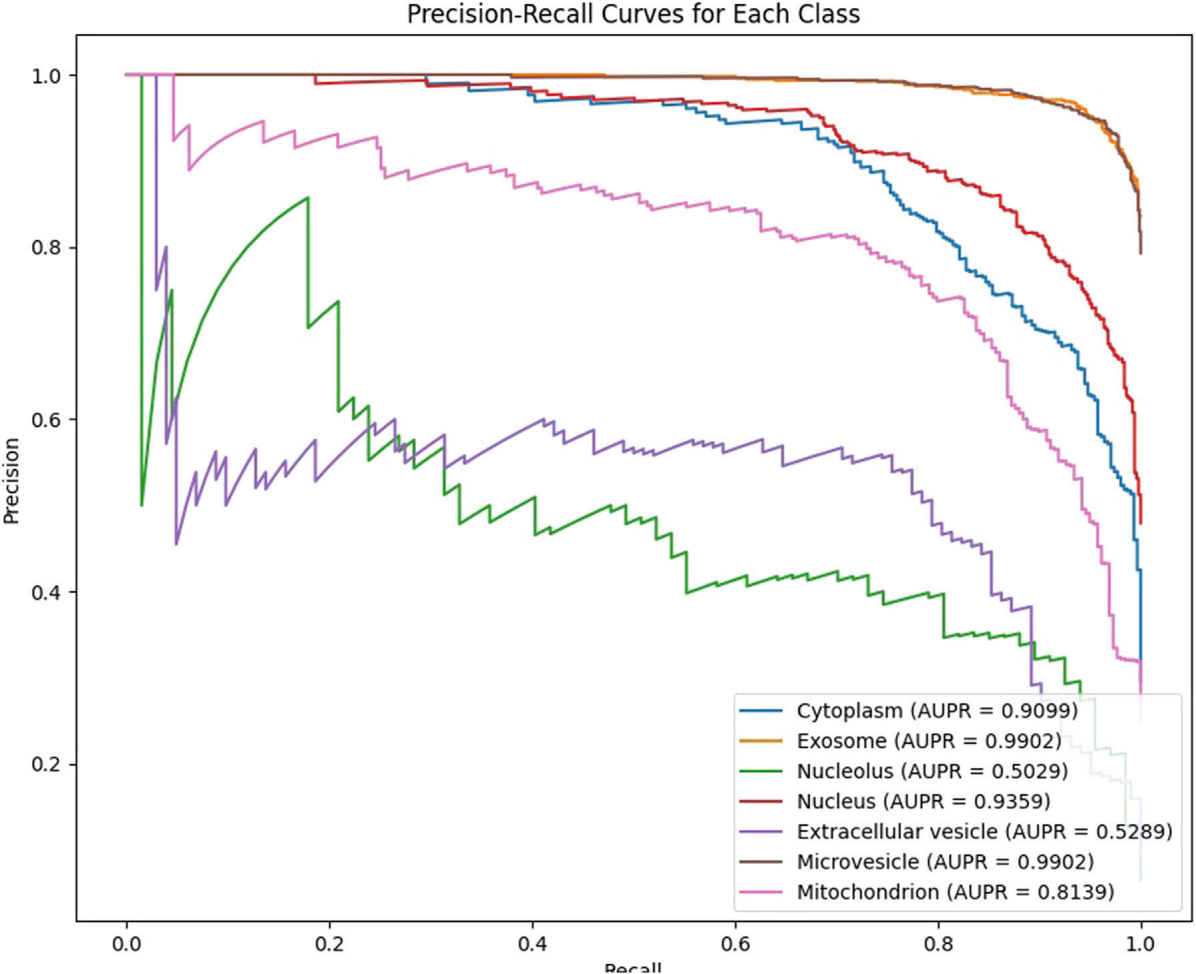
The Model GTMALoc 10-fold cross-validation AUPR value.

In our experiments, we employ 10-fold cross-validation to comprehensively assess the generalization ability of the model. The dataset is randomly shuffled and evenly divided into 10 subsets, each fold rotation assigned one decile to testing and nine to training, ensuring comprehensive parameter optimization. After training, the model generates predicted probabilities for each class on the test set, which are then mapped to the [0,1] range using the Sigmoid activation function and binarized with a threshold of 0.5. For each fold, we calculate the Area Under the ROC Curve (AUC) and the Area Under the Precision-Recall Curve (AUPR) as evaluation metrics, and record the results for each class. As shown in Figures 2, 3, our model achieves an average AUC of 0.9108 and an average AUPR of 0.8102 on the multi-label subcellular localization task, fully demonstrating the model’s effectiveness and robustness in capturing multimodal features and their high-order interactions.

### 8.2 Comparative experiments

To comprehensively evaluate the performance of the GTMALoc model, we use both 5-fold and 10-fold cross-validation strategies and systematically compare it with four existing methods (MiRLoc, MirLocPredictor, DAmiRLocGNet, and PMiSLocMF). The evaluation metrics include AUC and AUPR to thoroughly assess the model’s effectiveness.

As shown in Table 1, GTMALoc achieves an average AUC score of 0.9094 under 5-fold cross-validation, outperforming the other methods across most subcellular localization categories. It performs particularly well in structurally complex or sparsely connected categories such as cytoplasm (0.9240), extracellular vesicle (0.9115), and microvesicle (0.9113), demonstrating its strength in integrating high-dimensional heterogeneous information and modeling complex relationships. Table 2 presents the comparison based on AUPR, which primarily reflects the model’s robustness in class-imbalanced scenarios. GTMALoc also achieves the highest average AUPR of 0.8044, showing excellent performance in critical functional regions such as exosome (0.9900), nucleus (0.9248), and microvesicle (0.9900). Although the score slightly decreases in the nucleolus (0.5142), where signals are sparse, the overall performance remains superior.

**TABLE 1:** AUC Performance Comparison of miRNA Subcellular Localization Models Based on 5-Fold Cross-Validation.

Subcellular localization	MiRLoc	MirLocPredictor	DAmiRLocGNet	PMiSLocMF	GTMALoc
Cytoplasm	0.8356	0.5740	0.8601	0.8901	0.9240
Exosome	0.7391	0.5839	0.7043	0.9503	0.9251
Nucleolus	0.9080	0.5289	0.9280	0.9254	0.9271
Nucleus	0.7766	0.6755	0.7955	0.8745	0.8854
Extracellular vesicle	0.8001	0.6330	0.8317	0.8634	0.9115
Microvesicle	0.5093	0.5967	0.6729	0.9369	0.9113
Mitochondrion	0.7691	0.6742	0.8321	0.8689	0.8815
Average AUC	0.7625	0.6094	0.8035	0.9013	0.9094

**TABLE 2:** AUPR Performance Comparison of miRNA Subcellular Localization Models Based on 5-Fold Cross-Validation.

Subcellular localization	MiRLoc	MirLocPredictor	DAmiRLocGNet	PMiSLocMF	GTMALoc
Cytoplasm	0.7239	0.8472	0.7621	0.8149	0.8957
Exosome	0.9822	0.8219	0.9233	0.9897	0.9900
Nucleolus	0.4141	0.4901	0.5732	0.5199	0.5142
Nucleus	0.8111	0.4313	0.7945	0.8869	0.9248
Extracellular vesicle	0.2902	0.3464	0.4600	0.4682	0.5153
Microvesicle	0.9201	0.2443	0.8872	0.9854	0.9900
Mitochondrion	0.5189	0.3111	0.6867	0.7275	0.8009

Furthermore, as indicated in Table 3, GTMALoc’s average AUC increases to 0.9108 under 10-fold cross-validation, further confirming the model’s stability and generalization across different data splits. Its outstanding performance in categories such as nucleolus, cytoplasm, and exosome highlights its ability to accurately identify miRNAs localized in these regions. This advantage is largely attributed to the multi-head attention mechanism, which effectively captures complex sequence patterns and graph-structured information. As shown in Table 4, although GTMALoc’s AUPR scores for some sub-tasks are slightly lower than those of PMiSLocMF, the overall average AUPR reaches 0.8102, demonstrating strong resilience to data imbalance. We observe significant performance differences across localization categories: exosome and microvesicle achieve near-perfect AUPR scores, indicating successful recognition of key regional features, while performance in nucleolus and extracellular vesicle is relatively lower, likely due to insufficient positive samples and data sparsity. This suggests that future work should focus on improving data quality or adopting sample augmentation strategies to enhance performance in low-signal categories. Overall, the experimental results demonstrate that GTMALoc consistently exhibits strong predictive power and generalization ability under various cross-validation strategies, confirming its feasibility and practicality as a reliable tool for miRNA subcellular localization prediction.

**TABLE 3:** AUC Performance Comparison of miRNA Subcellular Localization Models Based on 10-Fold Cross-Validation.

Subcellular localization	MiRLoc	MirLocPredictor	DAmiRLocGNet	PMiSLocMF	GTMALoc
Cytoplasm	0.8366	0.5741	0.8606	0.8909	**0.9260**
Exosome	0.7395	0.5842	0.7051	**0.9513**	0.9177
Nucleolus	0.9085	0.5286	**0.9289**	0.9267	0.9258
Nucleus	0.7765	0.6752	0.7960	0.8764	**0.8916**
Extracellular Vesicle	0.8003	0.6335	0.8350	0.8574	**0.9193**
Microvesicle	0.5099	0.5973	0.6757	**0.9502**	0.9110
Mitochondrion	0.7694	0.6758	0.8332	0.8702	**0.8843**
Average AUC	0.7630	0.6098	0.8049	0.9033	**0.9108**

The bold values indicate the best result.

**TABLE 4:** AUPR Performance Comparison of miRNA Subcellular Localization Models Based on 10-Fold Cross-Validation.

Subcellular localization	MiRLoc	MirLocPredictor	DAmiRLocGNet	PMiSLocMF	GTMALoc
Cytoplasm	0.7258	0.8391	0.7636	0.8192	**0.9099**
Exosome	0.9892	0.8248	0.9248	**0.9905**	0.9902
Nucleolus	0.4148	0.4925	0.5739	**0.5298**	0.5029
Nucleus	0.8102	0.4349	0.7961	0.8763	**0.9359**
Extracellular Vesicle	0.2916	0.3434	0.4619	0.4695	**0.5289**
Microvesicle	0.9203	0.2469	0.8883	0.9866	**0.9902**
Mitochondrion	0.5277	0.3113	0.6882	0.7294	**0.8139**
Average AUPR	0.6689	0.4990	0.7281	0.7716	**0.8102**

The bold values indicate the best result.

### 8.3 Ablation study

**FIGURE 4:**
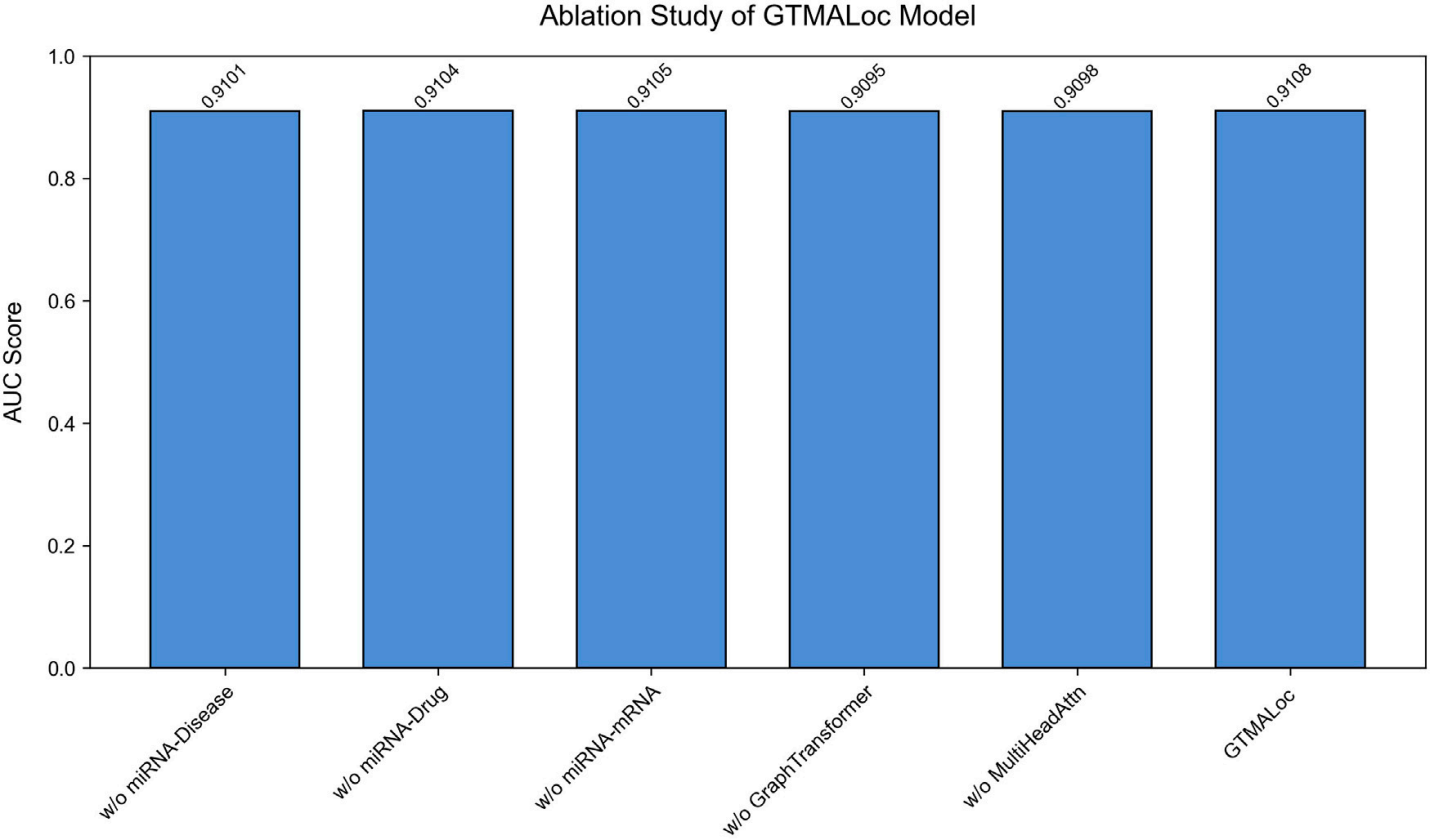
The Model GTMALoc ablation experiment.

To validate the contribution of each submodule within the overall architecture, we conduct detailed ablation studies by sequentially removing key components of the model and observing the resulting AUC performance on the multi-label subcellular localization task. As shown in Figure 4, we design five ablation settings: removing the miRNA-disease association network, removing the miRNA-drug interaction network, removing the miRNA-mRNA regulatory network, removing the graph transformer module, and removing the multi-head attention mechanism. All other modules remain unchanged across experiments to ensure a consistent model structure and fair evaluation. Each module contributes positively to the model’s overall performance, especially the graph transformer and multi-head attention modules, which play crucial roles in capturing high-order cross-modal interactions and both local and global structural features.

### 8.4 Parameter study

**FIGURE 5:**
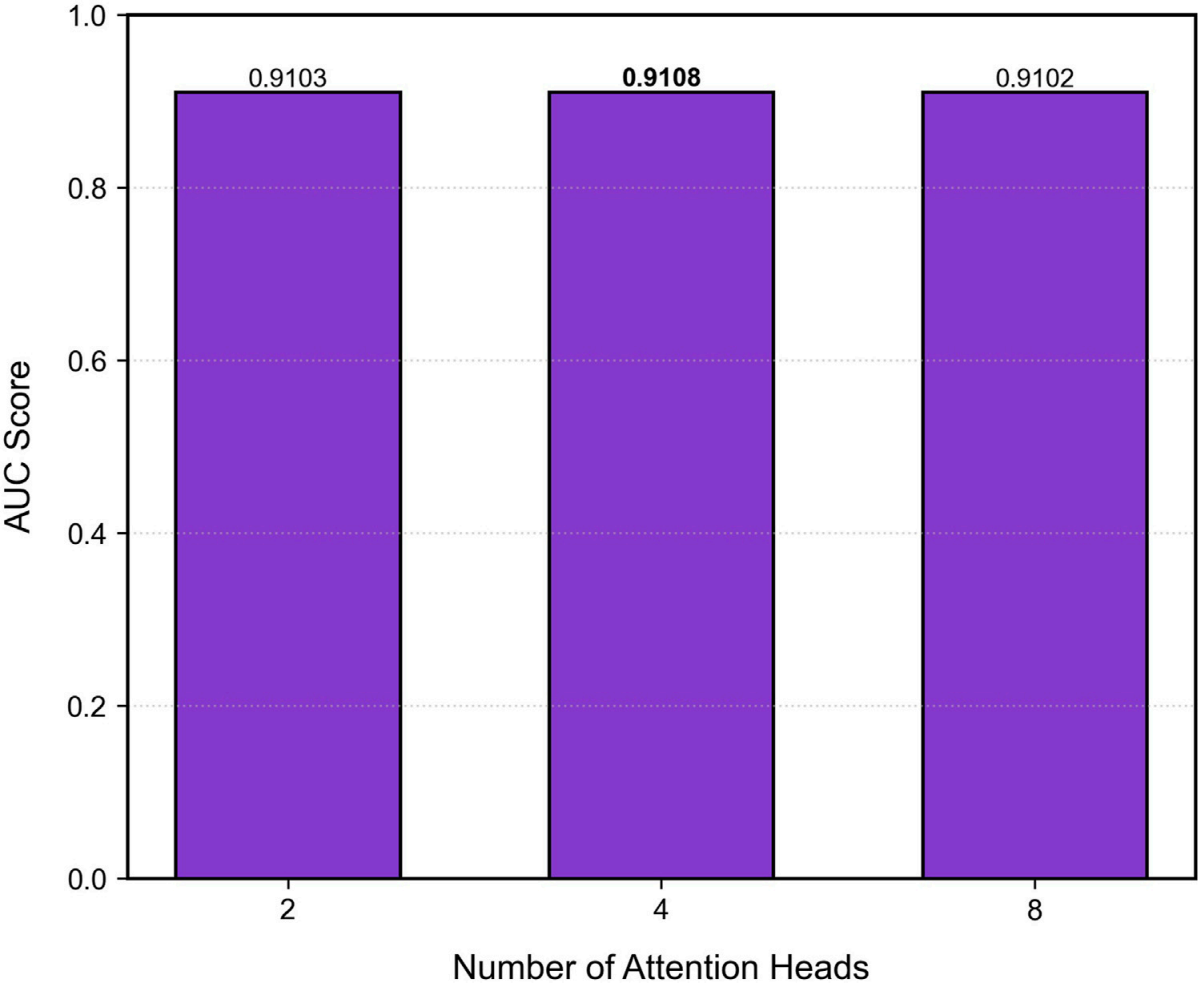
The Model GTMALoc parameter experiment.

To investigate the effect of the number of attention heads on model performance, we systematically evaluate different configurations (2, 4, and eight heads) on the validation set, as shown in Figure 5. A grid search strategy fixes other hyperparameters while varying the number of attention heads to observe sensitivity in the AUC metric. Results show that the model achieves peak performance (AUC = 0.9108) when the number of heads is set to 4, outperforming the 2-head and 8-head configurations by 0.0005 and 0.0006, respectively. This can be attributed to two main factors: (1) a moderate number of heads helps capture complementary interaction patterns in parallel subspaces, enhancing the model’s ability to fuse features across networks; (2) exceeding the optimal number of heads leads to redundancy in attention weights and an increased risk of local overfitting. Therefore, we adopt the 4-head configuration in the final architecture, balancing computational efficiency and predictive accuracy.

## 9 Case studies

To further demonstrate the practical utility of GTMALoc in predicting miRNA subcellular localization, we conduct case studies across seven subcellular categories: cytoplasm, exosomes, nucleolus, nucleus, extracellular vesicles, microvesicles, and mitochondrion. For each compartment, we select the top five miRNAs with the highest predicted probabilities generated by GTMALoc. We then manually verify these predictions against experimental evidence reported in the scientific literature. In total, 35 miRNA–localization associations are examined. As shown in Table 5 of these are supported by published studies, while only five lack current experimental validation.

**TABLE 5:** Case studies of miRNA subcellular localizations.

Rank	miRNA	Localization	Evidence	Rank	miRNA	Localization	Evidence
1	miR-122	Cytoplasm	PMID:34073601	4	miR-10a	Nucleus	PMID:30405557
2	miR-16	Cytoplasm	PMID:26304540	5	miR-26a	Nucleus	PMID:26304540
3	miR-34a	Cytoplasm	Unconfirmed	1	miR-142-3p	Extracellular vesicle	PMID:36277256
4	miR-146b-5p	Cytoplasm	PMID:37108595	2	miR-92a	Extracellular vesicle	PMID:22506055
5	miR-21	Cytoplasm	PMID:30405557	3	miR-221	Extracellular vesicle	PMID:28304367
1	miR-21	Exosome	PMID:29515311	4	miR-48	Extracellular vesicle	Unconfirmed
2	miR-126	Exosome	PMID:22506055	5	miR-155	Extracellular vesicle	PMID:22424232
3	miR-155	Exosome	PMID:22424232	1	miR-126	Microvesicle	PMID:34881308
4	miR-16	Exosome	PMID:22506055	2	miR-143	Microvesicle	PMID:31626610
5	miR-224	Exosome	PMID:30765428	3	miR-223	Microvesicle	PMID:31626610
1	miR-206	Nucleolus	PMID:19723800	4	miR-199a	Microvesicle	PMID:34881308
2	miR-340-5p	Nucleolus	PMID:19723800	5	miR-21	Microvesicle	PMID:31626610
3	miR-149	Nucleolus	PMID:31732639	1	miR-1	Mitochondrion	PMID:38205681
4	miR-21	Nucleolus	PMID:26674922	2	miR-365	Mitochondrion	PMID:19941672
5	miR-1	Nucleolus	Unconfirmed	3	miR-302a	Mitochondrion	PMID:19941672
1	miR-29b	Nucleus	PMID:26304540	4	miR-37	Mitochondrion	Unconfirmed
2	miR-320	Nucleus	PMID:26674922	5	miR-7	Mitochondrion	PMID:38205681
3	miR-9	Nucleus	Unconfirmed				

Taking miR-122 and miR-21 as representative examples, we analyze the alignment between GTMALoc’s predictions and reported biological findings. miR-122 is a liver-specific miRNA that is highly enriched in hepatocytes, and its cytoplasmic localization is well supported by experimental evidence Zhang et al. (2021). Previous studies indicate that miR-122 plays a crucial role in liver homeostasis by regulating lipid metabolism, cholesterol biosynthesis, and HCV replication through mRNA binding Ren et al. (2008). GTMALoc assigns a high confidence score of 0.98 for its cytoplasmic localization and captures its interactions with liver metabolism-related mRNA nodes, highlighting the model’s capacity to extract biologically meaningful features from the molecular network. In contrast, miR-21 is known for its multi-localization behavior and is highly expressed in various cancer types. It has been shown to be secreted via exosomes, contributing to immune modulation and tumor microenvironment remodeling Krishnamurthy et al. (2018), and also localizes in the nucleolus, where it may influence non-coding RNA processing Beckett et al. (2015). GTMALoc successfully predicts both localizations with high confidence and focuses attention on miR-21’s connections to tumor-associated signaling pathways, consistent with its known roles in cell proliferation, anti-apoptosis, and inflammatory response. These case studies suggest that GTMALoc not only achieves accurate subcellular localization predictions but also provides biologically interpretable outputs, particularly for multi-localized miRNAs, offering valuable insights for downstream functional analysis and subcellular mechanism exploration.

## 10 Conclusion

In this study, we propose a computational model, GTMALoc, for predicting miRNA subcellular localization. GTMALoc combines graph transformers with a multi-head attention mechanism to fuse heterogeneous biological information from multiple sources. Specifically, the model effectively integrates miRNA sequence features, interaction network structures, and functional properties. Through graph-based modeling and dynamic attention weighting, GTMALoc learns more discriminative high-dimensional feature representations, significantly improving the accuracy of localization prediction. We conduct a comprehensive evaluation of GTMALoc on public datasets. The results show that GTMALoc outperforms existing methods on multiple performance metrics, especially in handling sparse graph structures and high-dimensional feature spaces. Ablation studies confirm the key contributions of each feature modality and attention component to the model’s overall performance. Additionally, through representative case studies, we validate the biological interpretability of GTMALoc. The predicted subcellular localizations are not only consistent with known miRNA functions reported in the literature but also reveal potential regulatory modes that have not been fully explored. Given that miRNA localization is closely related to its regulatory roles in various cellular contexts, accurate localization prediction provides valuable insights into miRNA-mediated mechanisms under physiological and pathological conditions.

Although GTMALoc performs well in various experiments, it still faces some limitations. We integrate multiple heterogeneous features, such as sequence data, functional similarity, and molecular interaction networks; however, biological data often contain noise and incompleteness. For example, the functional annotations of many miRNAs remain incomplete, and some interaction networks may contain missing data or experimental biases, which can adversely affect the accuracy of feature learning. Additionally, differences among data sources in species, experimental conditions, or time points introduce biases and impair the model’s generalizability. In future work, we plan to further refine the model architecture to improve its interpretability, adaptability, and applicability in real-world biomedical research scenarios.

## Data Availability

Publicly available datasets were analyzed in this study. This data can be found here: The datasets for this study are openly available in the public domain: RNALocate v2.0 at http://www.rnalocate.org/ or http://www.rna-society.org/rnalocate/. The code and data that support the findings of this study are available at https://github.com/27167199/GTMALoc.
